# The inhibitor of apoptosis proteins antagonist Debio 1143 promotes the PD-1 blockade-mediated HIV load reduction in blood and tissues of humanized mice

**DOI:** 10.1371/journal.pone.0227715

**Published:** 2020-01-24

**Authors:** Michael Bobardt, Joseph Kuo, Udayan Chatterji, Norbert Wiedemann, Gregoire Vuagniaux, Philippe Gallay

**Affiliations:** 1 Department of Immunology & Microbiology, The Scripps Research Institute, La Jolla, California, United States of America; 2 Debiopharm International S.A., Lausanne, Switzerland; University Hospital Zurich, SWITZERLAND

## Abstract

The immune checkpoint programmed cell death protein 1 (PD-1) plays a major role in T cell exhaustion in cancer and chronic HIV infection. The inhibitor of apoptosis protein antagonist Debio 1143 (D1143) enhances tumor cell death and synergizes with anti-PD-1 agents to promote tumor immunity and displayed HIV latency reversal activity *in vitro*. We asked in this study whether D1143 would stimulate the potency of an anti-human PD-1 monoclonal antibody (mAb) to reduce HIV loads in humanized mice. Anti-PD-1 mAb treatment decreased PD-1+ CD8+ cell population by 32.3% after interruption of four weeks treatment, and D1143 co-treatment further reduced it from 32.3 to 73%. Anti-PD-1 mAb administration reduced HIV load in blood by 94%, and addition of D1143 further enhanced this reduction from 94 to 97%. D1143 also more profoundly promoted with the anti-PD-1-mediated reduction of HIV loads in all tissues analyzed including spleen (71 to 96.4%), lymph nodes (64.3 to 80%), liver (64.2 to 94.4), lung (64.3 to 80.1%) and thymic organoid (78.2 to 98.2%), achieving a >5 log reduction of HIV loads in CD4+ cells isolated from tissues 2 weeks after drug treatment interruption. *Ex vivo* anti-CD3/CD28 stimulation increased the ability to activate exhausted CD8+ T cells in infected mice having received *in vivo* anti-PD-1 treatment by 7.9-fold (5 to 39.6%), and an additional increase by 1.7-fold upon D1143 co-treatment (39.6 to 67.3%). These findings demonstrate for the first time that an inhibitor of apoptosis protein antagonist enhances in a statistically manner the effects of an immune check point inhibitor on antiviral immunity and on HIV load reduction in tissues of humanized mice, suggesting that the combination of two distinct classes of immunomodulatory agents constitutes a promising anti-HIV immunotherapeutic approach.

## Introduction

WHO and UNAIDS estimated that 40 million people live with HIV. The Centers for Disease Control and Prevention estimated that 38,500 people were newly infected with HIV in the United States in 2015, and 2.1 million worldwide [1]. T cells have a critical function in constraining viremia during acute and chronic HIV infection. CD8+ T cells are responsible for the rapid decrease of viremia during acute HIV infection [2–4]. CD8+ T cells inhibit HIV replication *in vitro* [5], and CD8+ T cell depletion in SIV-infected primates resulted in a loss of viremia control during infection [6]. CD8+ T cells control viremia via cytotoxic activities [6] and the production of soluble factors such as CCR5 chemokine ligands [5, 7–12]. However, AIDS progression during sustained chronic infection often leads to impairment and exhaustion of effector and memory CD8+ T cells, resulting in a boost of viremia [13]. CD8+ T cell exhaustion was observed during chronic lymphocytic choriomeningitis virus (LCMV) infection in mice where LCMV-specific CD8+ T cells exhibited diminished abilities to both eliminate infected cells and produce antiviral cytokines [13]. Dysfunctional CD8+ T cells were found in humans during chronic HIV, hepatitis B virus (HBV), hepatitis C virus (HCV) and human T lymphotropic virus (HTLV) infections as well as in primates during chronic SIV infection [14].

The immune checkpoint programmed cell death protein 1, also known as PD-1 or CD279 (cluster of differentiation 279) is highly expressed on exhausted CD8+ T cells in chronically LCMV-infected mice [15]. Neutralizing PD-1 with anti-PD-1 monoclonal antibodies or its ligand PD-L1 profoundly increased LCMV-specific T cell activities and expansion resulting in a profound decrease in viral load [15]. Importantly, the PD-1/PD-L1 pathway controls the dysfunction of CD8+ T cells during chronic HIV infection [16–18]. High PD-1 expression on exhausted HIV-specific CD8+ T cells correlates with elevated viral load and reduced CD4+ T cell numbers. *Ex vivo* neutralization of the PD-1/PD-L1 pathway results in HIV-specific CD8+ T cell multiplication and TNFα, IFNγ and the serine protease granzyme B release, suggesting a reconstitution of effector functions of CD8+ T cells [16–18]. Neutralization of the PD-1/PD-L1 pathway in chronically infected macaques not only led to SIV-specific CD8+ T cell proliferation with restored effector functions, but also to both a decrease in viral load and extended survival [19].

PD-1 also plays a major role in mediating T cell exhaustion in cancer [20–29]. Importantly for the present study, the pro-apoptotic and immunotherapeutic agent D1143 promotes the anti-tumor effect of anti-PD-1/PD-L1 agents [30–31]. D1143 is an inhibitor of apoptosis protein antagonist (IAPa), which induces apoptotic cell death and blocks pro-survival signaling in cancer cells, by triggering the degradation of inhibitor of apoptosis proteins (IAP) and activation of the non-canonical NF-kB signaling pathway [32]. IAPa mimic the structure of a tetrapeptide sequence from second mitochondria-derived activator of caspases (SMAC) to bind to the common baculoviral IAP repeat (BIR) domain of members of the IAP protein family, including XIAP, BIRC2 and BIRC3 [33–35]. IAPa binding modulates the ubiquitin ligase function of these IAP members [33–35]. We recently reported that the IAPa D1143 modulates the non-canonical NF-kB pathway by rapidly degrading a repressor of this important signaling pathway—the baculoviral IAP repeat-containing 2 (BIRC2) [36].

IAP were first identified as promoters of cancer cell survival by regulating the NF-κB pathway and are now known as critical regulators of multiple pathways that control cell death, proliferation and differentiation [37]. Importantly, IAPa reverse this effect, a property currently tested in multiple clinical studies for the treatment of hematological and solid cancers in combination with radio- and/or chemo-therapy and ICI [38]. More recently, IAP were found to regulate the innate immunity, especially Toll-like (TLR), NOD (nucleotide-binding oligomerization domain-like), NLR (NOD-like) and retinoic acid-inducible gene I (RIG-1)-like receptor signaling [32]). IAP were also found to control the adaptive immunity including B cell proliferation and survival, T cell response to antigenic peptides and tumor antigens, and monocyte and dendritic cell development and activation [39–43]). Altogether these findings suggest that IAPa have great potential as immunotherapeutic agents against both pathogens and cancers [37].

D1143 has shown promising antitumor activities as a single agent as well as in combination with different treatment modalities including conventional chemotherapy or radiation, targeted agents, as well as immunotherapies [44–47]. Of note, D1143 augments the tumor-specific adaptive immunity induced by ablative radiation therapy, while reducing host immunosuppressive cell infiltrates in the tumor microenvironment in a TNFα, IFNγ and CD8^+^ T-cell-dependent manner [48]. D1143 enhanced CD4+ and CD8+ intracellular IFNγ expression in a concentration-dependent manner following *ex vivo* [30] anti-CD3/CD28 stimulation. An effect that was further increased in presence of the anti-PD-1 mAb nivolumab [30]. In MBT-2 tumor-bearing mice, the combination of D1143 and an anti-PD-L1 mAb decreased tumor growth and increased survival [30]. This synergy will be further explored in a phase-Ib dose-finding clinical study combining D1143 and Avelumab (anti-PD-L1 mAb) in patients with advanced solid malignancies and non-small cell lung cancer (CT# 03270176) [49]. Since D1143 enhances the beneficial effect of PD-1 neutralization in various cancers, we asked in this study whether D1143 would also enhance the anti-HIV immunity of an anti-PD-1 mAb by restoring the capacity of exhausted CD8+ cells to kill infected cells, and impact HIV loads in blood and tissues of humanized BLT mice.

## Materials and methods

### Drugs and antibodies

D1143 was obtained from Debiopharm International S.A., anti-human PD-1 mAb used for BLT mouse treatment was obtained from Bio X Cell (Clone J116), anti-human PD-1 mAb (Clone EH12-1540-29C9) used for cell surface staining was obtained from Synagis [50], and anti-human CD8 (Clone RPA T8), CD3 (Clone UCHT1), CD4 (clone OKT4), CD45 (Clone HI30) and IFNγ (Clone 4S.B3) antibodies used for cell staining were obtained from BioLegend. Note that anti-human PD-1 antibodies Clone J116 and Clone EH12-1540-29C9 recognize distinct epitopes.

### Animal care

Animal housing: individually ventilated cage (IVC) racks are used to house the majority of mice. HEPA-filtered air is supplied into each cage at a rate of 60 air changes per hour. Mice are housed in solid bottom cages. Static mouse cages are changed at least once a week. Mouse individually ventilated cages (IVCs) are changed at least once every14 days. Certain strains of rodents (e.g., diabetic) are changed into clean cages more frequently as needed.

Room environment: heating, ventilation and air conditioning performance is routinely assessed as part of facility renovations, system repairs, and at least once every 3 years. Each animal room is equipped with a high/low thermo-hygrometer and its own computerized controlled thermostat. Animal care staff monitor and record animal room high/low temperatures and humidity daily on the room activity log. Temperature settings are consistent with Guide recommendations and are calibrated by the Engineering Department. Alarm points are set at ± 4°F. High or low-temperature alarms are annunciated to the engineer on duty 24 hours a day. DAR management is notified of excursions. Most of the animal facilities are also equipped with an Edstrom Industries Watchdog environmental monitoring system in addition to the automated building management system (BMS). The Watchdog system registers temperature and humidity and also sends alarms to Animal Resources management personnel. Humidity levels are not controlled in any of the facilities but are reliably maintained between 30–70% most of the year.

Diet: Food (Teklad LM-485 autoclavable diet) is provided ad lib to mice in wirebar lids.

Water: the IMM animal facility is equipped with a reverse osmosis (R/O) water purification system and automatic watering distribution system from Edstrom Industries. DAR receives monthly water quality reports from the City of San Diego. R/O purified water is monitored daily during the workweek. A number of parameters are monitored including conductivity, temperature, pH level and chlorine concentration. Automatic water delivery systems (room and rack distribution lines) are timed for daily in-line flushing. Quick disconnect drinking valves are sanitized with each cage change or more often if needed. System sanitation and preventive maintenance is performed by the DAR equipment technicians.

Acclimation period: mice are allowed up to 72 hours to stabilize into their new housing environment. Some experimental paradigms involve examining the behavioral response to novelty and therefore the animal cannot be habituated to the procedure.

Animal suffering: In order to minimize suffering, all surgical procedures are carried out under anesthesia using isoflurane (1–4%) in conjunction with ketamine/xylazine ip (90–120 mg/Kg and 10 mg/Kg). Mice are monitored every 15 minutes after induction for respiratory and heart rates if the surgical procedure requires more time. Animals are provided buprenorphine (0.05–2.5 mg/Kg s.c.) for 6–12 h followed by flunixine meglumine (2.5 mg/Kg s.c.) as a post-operative analgesic for 2 days post-implantation. Mice are observed 2 h, 6 h and 24 h post-surgery with daily monitoring during the course of the study. Mice are supplied with acidified water supplemented with sulfamethoxazole (or, sulfadiazine) with trimethoprim at a final concentration of 0.65–1.6 mg/mL to reduce chances of opportunistic bacterial colonization. Fetal human liver and thymus tissues were purchased from Advanced Bioscience Resources, Inc., Alameda, CA. Humanized mice were maintained at the Department of Animal Resources (DAR) at The Scripps Research Institute (TSRI) in accordance with protocols approved by the TSRI Ethics Committee, the Institutional Animal Care and Use Committee (Permit Number: 13–0001).

### Generation of Humanized BLT Mice

Humanized BLT mice (32 animals) were generated as described previously [50–55], by implanting 1-mm^3^ pieces of human fetal liver and thymus tissues (Advanced Bioscience Resources) under the kidney capsule in 6 to 8-week-old female NSG mice (Jackson Laboratories) bred at The Scripps Research Institute (TSRI). The cohort was produced with tissues from a single donor. CD34+ HSPC were purified from autologous fetal liver tissue, isolated by magnetic bead selection for CD34+ cells (Miltenyi), phenotyped cytometrically [50–55], and cryopreserved until injection (200,000–350,000 CD34+ cells) into mice 3 weeks after Thy/Liv implantation. Human reconstitution in peripheral blood was verified by flow cytometry as described previously [50–55]. Mice were maintained at the Department of Animal Resources (DAR) at TSRI in accordance with protocols approved by the TSRI Ethics Committee, the Institutional Animal Care and Use Committee (Permit Number: 13–0001). This study was carried out in strict accordance with the recommendations in the Guide for the Care and Use of Laboratory Animals of the National Institutes of Health. All surgery was performed under sodium pentobarbital anesthesia, and all efforts were made to minimize suffering. The method of sacrifice used for the experimental mice is cervical dislocation.

### HIV Infection of Humanized BLT Mice and Viral Load Quantification in Blood and Tissues

Stocks of HIV JR-CSF were prepared as previously described [50–55] and standardized by p24 ELISA. Humanized BLT mice were challenged i.v. with HIV JR-CSF (100 ng of p24 or 10^4^ Median Tissue Culture Infectious Doses (TCID_50_). We used a “simple randomization” for the 4 arms by choosing randomly 8 mice per arm. Three weeks post-HIV challenge, infection was confirmed by quantifying viral RNA by PCR viral load in peripheral blood (plasma) using one-step reverse transcriptase quantitative real-time PCR (qRT-PCR) (ABI custom TaqMan Assays-by-Design) according to the manufacturer’s instructions. Primers were 5-CATGTTTTCAGCATTATCAGAAGGA-3 and 5-TGCTTGATGTCCCCCCACT-3, and MGB-probe 5-FAM-CCACCCCACAAGATTTAAACACCATGCTAA-Q-3, where FAM is 6-carboxyfluorescein as we recently described [51–52]. The assay sensitivity was of 423 RNA copies per mL of plasma. For quantification of HIV RNA loads in tissues, RNA was extracted from at least 2x 10^6^ CD4+ cells isolated from the harvested tissues using EasySep^™^ Human CD4+ T Cell Isolation Kit (STEMCELL Technologies) and the RNeasy Mini Kit (Quiagen) and viral loads quantified by qRT-PCR as described above.

### FACS analyses

Ficoll-Hypaque density gradient centrifugation-derived PBMCs from blood collected from HIV-1-infected BLT mice were stained with conjugated anti-human CD8 and PD-1 antibodies. Expression of PD-1 in total gated CD8-positive cells was analyzed by FACS on a Novocyte 3000 flow cytometer (ACEA Biosciences). The primary data analysis was performed using NovoExpress.

### *Ex vivo* human CD8 T Cell Activation Analysis

At the end of week 16 –end of the four treatments–the percentage of human CD3+ and CD45+ cells in blood of HIV-infected BLT mice was quantified and isolated by FACS. CD45+ cells were isolated by EasySep^™^ Human CD45^+^ Cell Enrichment Kit (STEMCELL Technologies). Isolated human CD45+ cells (100,000) (Clone HI100, BioLegend) were stimulated *ex vivo* for 24 h with anti-CD3 (clone UCHT1, BioLegend) (200 ng/mL) and anti-CD28 (Clone CD28.2, BioLegend) (500 ng/mL) antibodies and the percentage of IFNγ+ CD8+ cells from gated total CD8+ cells (Clone RPA-T8, BioLegend) was quantified by FACS. Intracellular IFNγ staining (Clone 4S.B3, BioLegend) was performed according to the manufacturer’s instructions using Cytofix/Cytoperm Kit (BD Biosciences), which saponin-permeabilized and fix cells prior to staining.

### Statistical analysis

Percentages of cells and HIV viral load among different groups were evaluated by analysis of variance, followed by Bonferroni's multiple comparison tests through Prism (GraphPad Software; San Diego, CA). The alpha level was set at 0.05. Data are presented as the mean ± standard error, with indicated p-values from Bonferroni's multiple comparison tests.

## Results

### D1143 and anti-PD-1 mAb overcome human CD8+ T cell exhaustion

**Fig 1:**
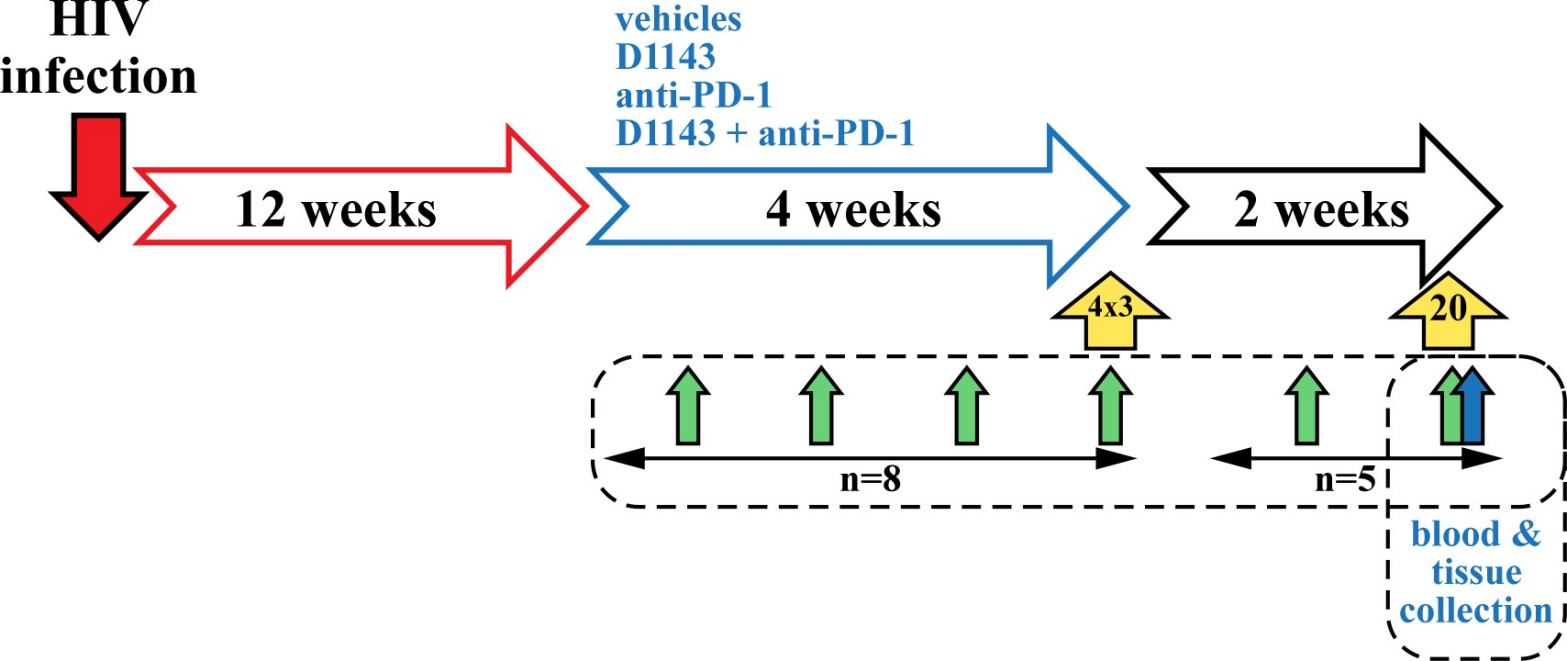
Experimental design for D1143 and anti-PD-1 mAb treatments and blood and tissue analyses in HIV-infected BLT mice.

Twelve weeks after infection with HIV-1, thirty-two BLT mice were split in 4 groups (n = 8), treated with four regimens and analyzed for 4 weeks (Fig 1). Group A received both vehicles. Group B received D1143 (100 mg/kg; QD1-5, p.o.) for 4 weeks (D1143 was given 5 days a week for 4 weeks). Group C received the anti-PD-1 mAb (8 doses of 200 μg, i.p., BIW) for 4 weeks. Group D received the combination of D1143 (100 mg/kg; QD1-5, p.o.) together with the anti-PD-1 mAb (200 μg/dose; BIW) for 4 weeks. The vehicle for the anti-PD1 antibody was PBS and the vehicle for D1143 is malic acid with sodium acetate, pH 4.5. In order to facilitate the interpretation of the data, we examined as an initial step the effect on viral loads reduction by the combination of D1143 and anti-PD-1 mAb in HIV-infected BLT mice. Body weight and HIV loads were analyzed at selected weeks. After the 4 weeks of treatment, three mice per group were sacrificed and immunologic markers assessed *ex vivo* by FACS (PD-1 expression on CD8+ T cells, intracellular IFNγ expression on anti-CD3/CD28 antibody-stimulated CD8+ T cells, CD3, CD45). The remaining mice (4x5 = 20) continued to be analyzed BIW for body weight, HIV loads and PD-1 expression on CD8+ T cells for two additional weeks. At the end of the study, animals were sacrified and viral RNA loads from human CD4+ cells in tissues (spleen, thymic organoid, lung, spleen, lymph nodes and liver) were quantified by qPCR as previously described [51–52].

**Fig 2:**
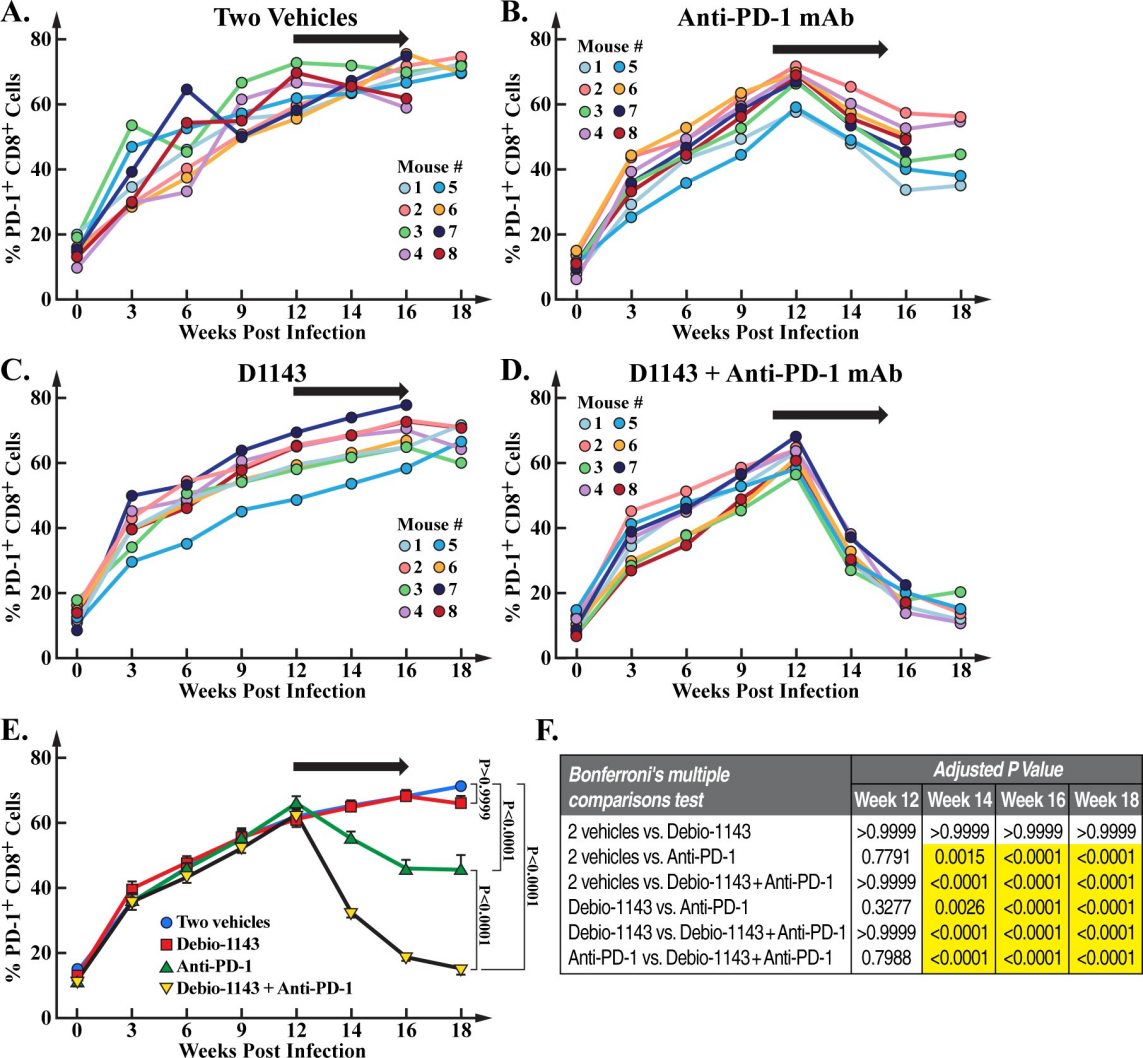
PD-1 expression on human CD8+ T cells from HIV-infected BLT mice. Blood was collected at week 0, 3, 6, 9, 12 (beginning of treatments), 14, 16 (end of treatments) and 18 (time of animal sacrifice) and isolated PBMCs stained with directly conjugated anti-human CD8 and anti-human PD-1 antibodies and analyzed by FACS. Data (median value of 8 mice per group/treatment and median value of 5 mice per group/treatment from week 16 to 18) are presented as percentage of human PD-1+ CD8+ T cells.

Previous studies demonstrated that HIV infection induces elevated PD-1 expression on human CD4+ and CD8+ T cells of humanized mice [50, 55–57] or infected individuals [25–29]. Thus, we examined PD-1 expression levels on human CD8^+^ T cells during HIV-1 infection of our humanized BLT mice. We confirmed that PD-1 expression increases over time (week 0 to 18) during HIV infection in vehicle-treated mice (Fig 2) [56]. We found that anti-PD-1 mAb 4-week treatment (from week 12 to 16) reduced PD-1 expression on human CD8+ T cells. Note that we analyzed the respective percentages of human CD4+ and CD8+ T cells among human CD45+ cells at day 0 and 14 (beginning and end of treatments). As previously described [56], the percentage of human CD4+ cells decreased in vehicle- (17.7 to 11.4%) or D1143-treated mice (17.2 to 10.8%) likely due to the HIV-1 infection-mediated depletion of human CD4+ cells while the percentage of human CD4+ cells slightly increases in anti-PD-1- (17.7 to 19.2%) and anti-PD-1/D1143-treated mice (18.1 to 20.2%). The percentage of human CD8+ cells remained relatively stable during the short two weeks of treatments in vehicle- (7.8 to 8.1%), anti-PD-1- (7.6 to 7.9%), D1143- (7.7 to 7.5%) and anti-PD-1/D1143-treated mice (7.6 to 8%). As previously reported, the anti-PD-1 treatment did not influence PD-1 expression on CD4+ T cells or CD4+ T cell counts [50]. D1143 alone has no effect on PD-1 expression on human CD8+ T cells (Fig 2). However, when combined with anti-PD-1 mAb, D1143 amplified the reduction of PD-1 surface expression on human CD8+ T cells mediated by the anti-PD-1 mAb treatment (Fig 2). Specifically, the anti-PD-1 mAb treatment decreased PD-1+ CD8+ cell population by 32.3% after four weeks of treatment, and D1143 co-treatment further reduced it from 32.3 to 73%. The enhancement of the anti-PD-1-mediated effect by D1143 is statistically significant (Fig 2F). The reduction of the percentage of PD-1-positive cells may arise from an expansion of PD-1-negative cells, a decrease in PD-1 levels or both. The raw data are presented in S1 Appendix.

### D1143 enhances the anti-PD-1 mAb-mediated reduction in HIV loads in blood and tissues

**Fig 3:**
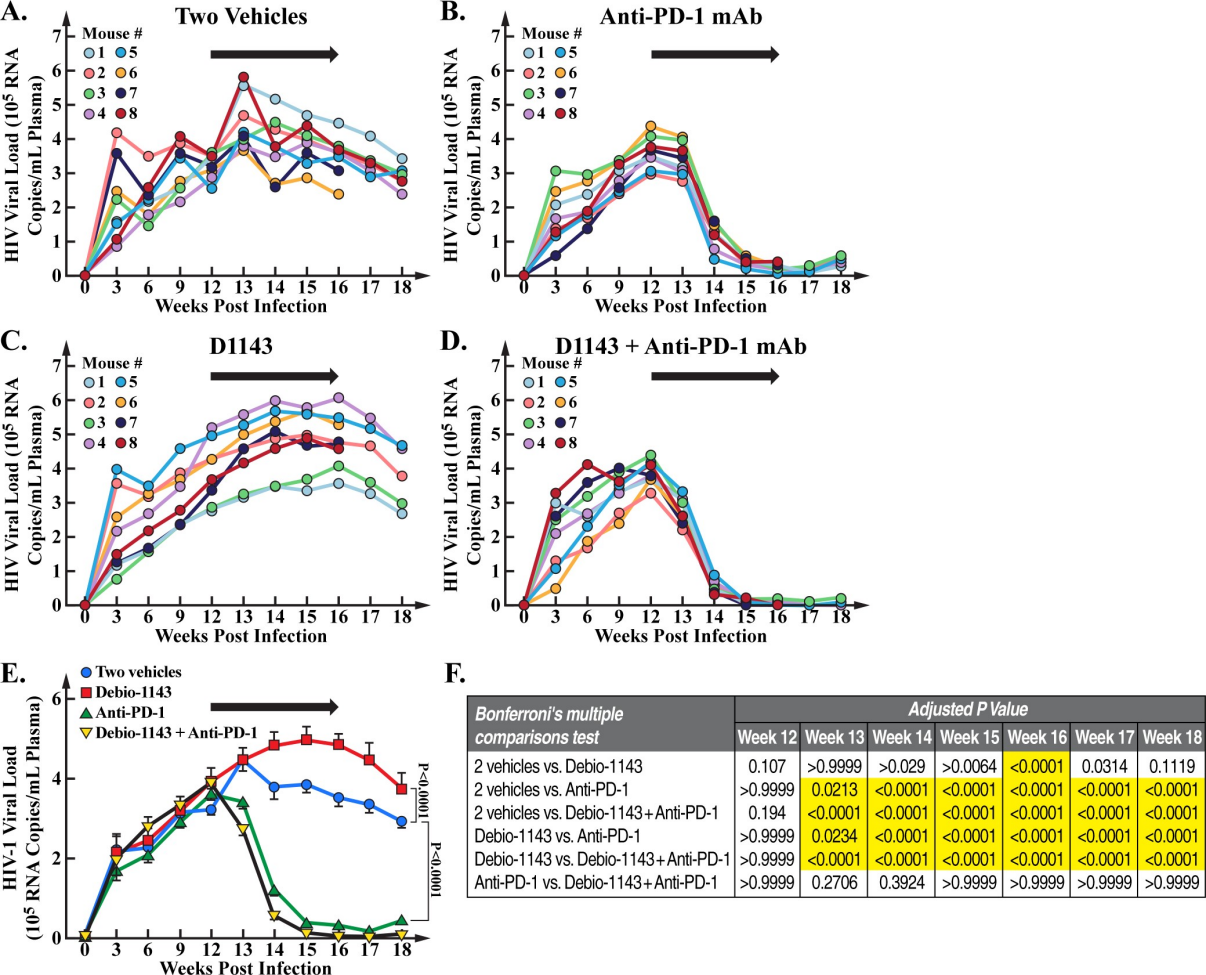
HIV loads in blood of HIV-infected BLT mice. Blood was collected at week 0, 3, 6, 9, 12 (beginning of treatments), 14, 16 (end of treatments) and 18 (time of animal sacrifice). HIV RNA levels in plasma were quantified using one-step reverse transcriptase quantitative real-time PCR as described previously [51–52]. Data (median value of 8 mice per group/treatment from week 0 to 16 and median value of 5 mice per group/treatment from week 16 to 18) are presented as HIV RNA copies per mL of plasma.

**Fig 4:**
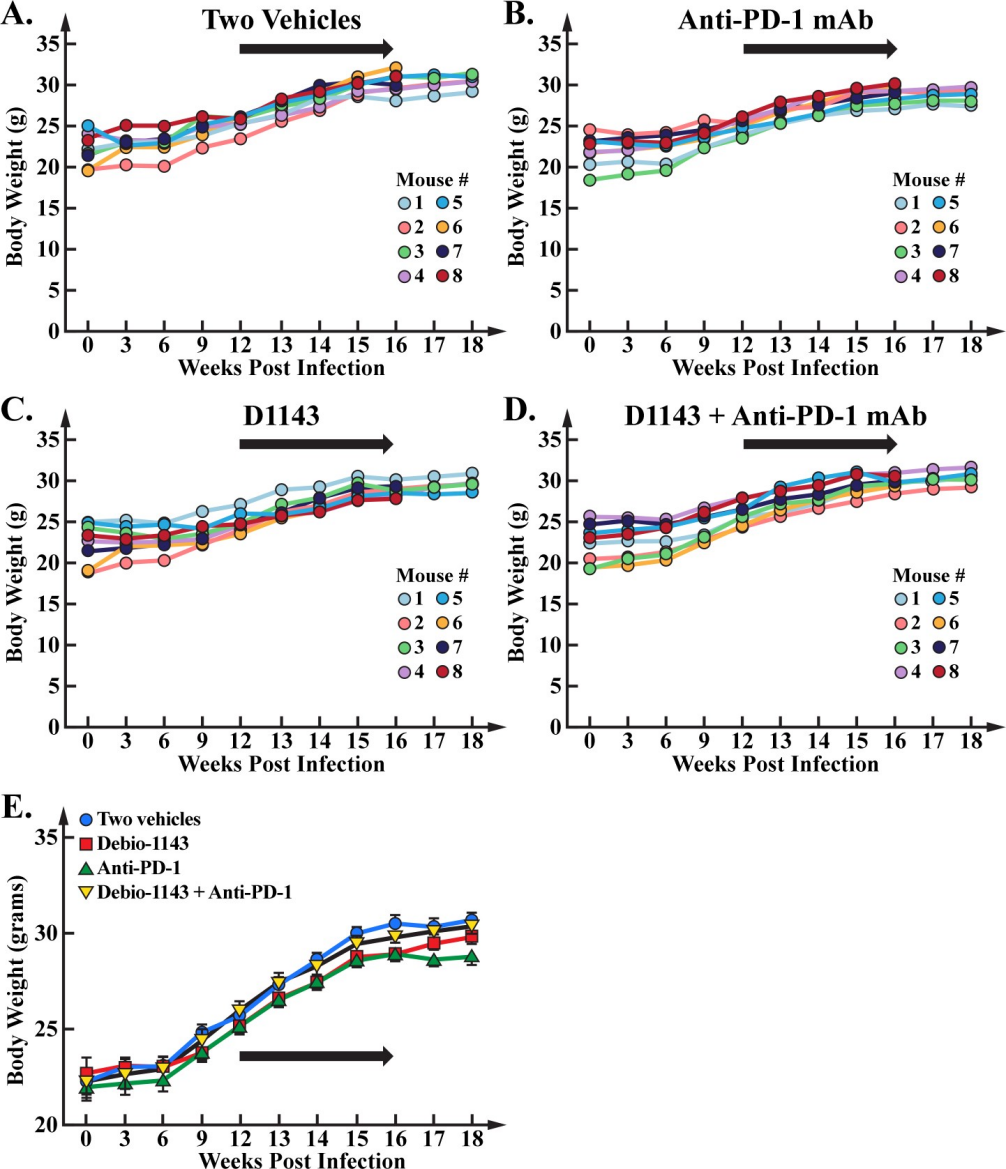
Body weight of HIV-infected BLT mice. Humanized mice were weighted at week 0, 3, 6, 9, 12 (beginning of treatments), 14, 16 (end of treatments) and 18 (time of animal sacrifice). Data (median value of 8 mice per group/treatment from week 0 to 16 and median value of 5 mice per group/treatment from week 16 to 18 and) are presented as HIV RNA copies per mL of plasma.

As previously reported [50, 55], the anti-PD-1 mAb treatment profoundly reduced blood HIV-1 loads (Fig 3B) compared to the vehicle treatment (Fig 3A). D1143 treatment enhanced blood HIV load compared to the vehicle treatment (Fig 3C) likely by enhancing viral transcription as we described previously [36]. D1143 amplified the anti-PD-1 mAb-mediated suppression of blood HIV loads at week 13, 14 and 15 (Fig 3D) although the D1143 enhancement of the anti-PD-1 treatment was not statistically significant (Fig 3F). Specifically, the anti-PD-1 mAb administration reduced HIV load in blood by 94%, and addition of D1143 further enhanced this reduction from 94 to 97%. The raw data are presented in S2 Appendix. None of the treatments affected general mouse health although some weight changes were observed (Fig 4). The raw data are presented in S3 Appendix.

**Fig 5:**
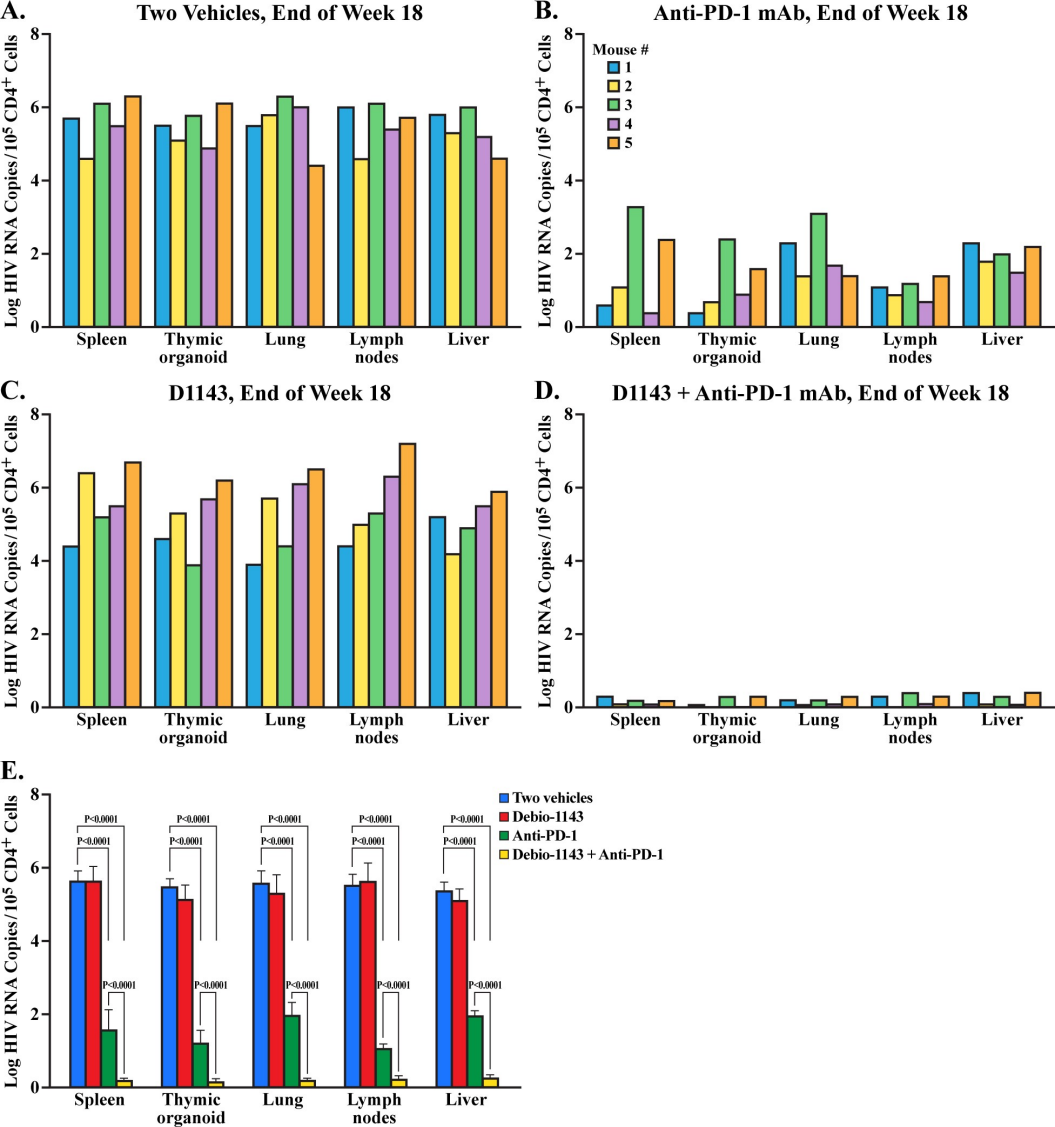
HIV loads in tissues of HIV-infected BLT mice. Selected tissues were collected at week 18 (time of animal sacrifice). HIV RNA levels in isolated human CD4+ cells were quantified using one-step reverse transcriptase quantitative real-time PCR as described previously [51–52]. Data (median value of 5 mice per group/treatment) are presented as HIV RNA copies per 100,000 isolated CD4+ cells.

At the end of the experiment (week 18), the five remaining mice per treatment group were sacrificed and HIV RNA levels in CD4+ cells from collected tissues were quantified by qPCR. Elevated HIV loads were detected in spleen, thymic organoid, lung, lymph nodes and liver from vehicle-treated (Fig 5A) or D1143-treated mice (Fig 5B). The anti-PD-1 mAb treatment significantly reduced HIV loads in tissues compared to vehicle and D1143 treatments (Fig 5C), suggesting a correlation between blood and tissue HIV loads. Importantly, D1143 once more enhanced the anti-PD-1 mAb mediated viral load suppression in all organs (Fig 5D) in a statistically manner (Fig 5E). Thus, D1143 more profoundly promoted with the anti-PD-1-mediated reduction of HIV loads in tissues than blood including spleen (71 to 96.4%), lymph nodes (64.3 to 80%), liver (64.2 to 94.4), lung (64.3 to 80.1%) and thymic organoid (78.2 to 98.2%), achieving a >5 log reduction of HIV loads in CD4+ cells isolated from tissues 2 weeks after drug treatment interruption. The raw data are presented in S4 Appendix.

### D1143 enhances the anti-PD-1 mAb-mediated activation of effector CD8+ T cells

**Fig 6:**
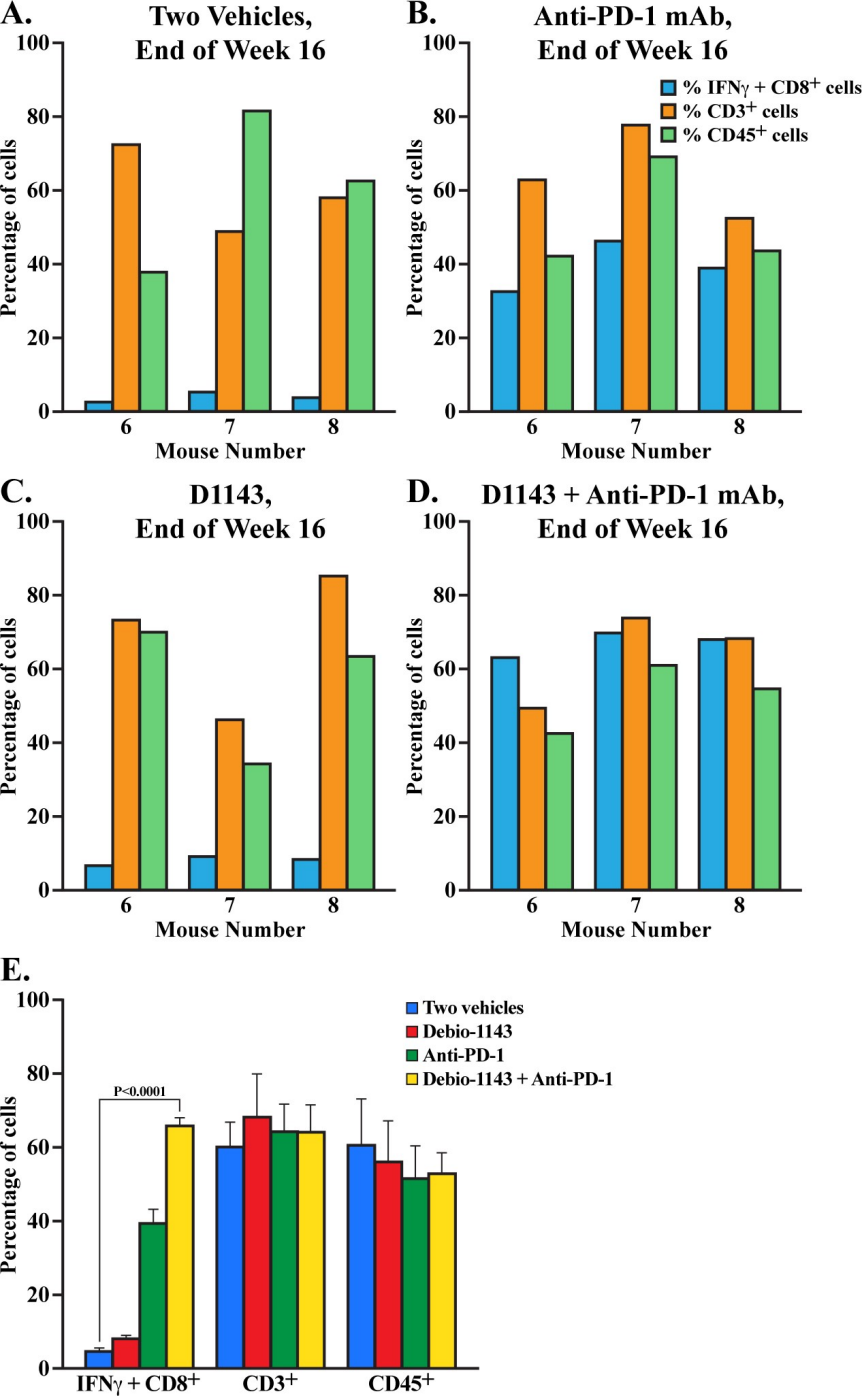
*Ex vivo* IFNγ production by human CD8^+^ effector T cells isolated from HIV-infected BLT mice. At the end of week 16 –end of the four treatments–percentages of human CD3+ and CD45+ cells in blood of HIV-infected BLT mice were quantified by FACS. Isolated human CD45+ cells were stimulated *ex vivo* for 24 h with anti-CD3 and anti-CD28 antibodies and the percentage of IFNγ+ CD8+ cells was quantified by FACS. Data (median value of 5 mice per group/treatment) are presented as percentages of CD3+, CD45+ and IFNγ+ CD8+ cells.

We examined at the end of week 16 –end of four treatments (beginning week 12 to end week 16) whether anti-PD-1 mAb treatments restored effector functions of CD8+ T cells, especially IFNγ production upon activation by CD3/CD28 antibody stimulation. We analyzed in three HIV-1-infected mice per treatment group percentages of blood CD3+ cells, CD45+ cells as well as percentage of IFNγ+ CD8+ cells after *ex vivo* stimulation of CD45+ cells for 24 h with anti-CD3 and anti-CD28 antibodies. Levels of human CD3+ and CD45+ cells were similar between the four treatment groups (Fig 6). We analyzed the percentages of total CD45+ human cells and double positive CD45+ CD3+ human T cells. We found that after *ex vivo* anti-CD3/CD28 antibody exposure, the percentage of IFNγ+ levels in CD8+ T cells derived from anti-PD-1 mAb-treated HIV-1-infected mice (Fig 6B) were superior to those derived from vehicle- (Fig 6A) and D1143-treated mice (Fig 6C). Importantly, D1143 enhanced the anti-PD-1 mAb-mediated enrichment in IFNγ+ CD8+ cells (Fig 6D) although not in a statistically manner. Specifically, the anti-PD-1 treatment increased the activation of human CD8+ T cells isolated from humanized mice using IFNγ as activation marker by 7.9-fold (5 to 39.6%), and an additional increase by 1.7-fold upon D1143 co-treatment (39.6 to 67.3%). The raw data are presented in S5 Appendix. This finding suggests that the combination of D1143 with anti-PD-1 mAb restored the effector functions of human CD8+ T cells in HIV-infected BLT mice and that the reduction of HIV loads in blood and tissues could be explained in part due to an enhancement of the CD8+ T cell-mediated antiviral immune response by the combination of the two distinct classes of immunomodulators, IAPa and ICI.

## Discussion

Testing new therapeutic approaches to reduce or even eliminate these HIV cellular reservoirs is of critical clinical importance. One attractive approach would be to reinvigorate the exhausted immune system during chronic infection, especially CD8+ T cells, in order to restore their ability to recognize and kill latently infected CD4+ cells. Effector antiviral CD8+ T cells possess several functional properties including cytokine (IFNγ, TNFα, IL-2, etc.) production, cytotoxic potential (i.e., perforin, granzymes), high proliferative potential, and low apoptosis [58]. CD8+ T cell exhaustion is a feature of chronic infections (HIV, HBV, HCV, etc.) in primates and humans [23–24, 26, 58]. Previous work suggests that PD-1 plays a key role in the exhaustion of virus-specific CD8+ T cells during HIV infection [23–24, 26, 58]. PD-1 expression is high on HIV-specific CD8+ T cells and that that relief of exhaustion through PD-1/PD-L1 leads to increased CD8+ T cell proliferation and effector molecule production, suggesting an overall increase in effector function [23–24, 26, 58]. Altogether these findings suggest that PD-1 blockade represents an attractive approach to reinvigorate exhausted HIV-specific CD8+ T cells and reinstate their capacity to kill infected CD4+ cells. In this study, we tested a novel strategy with the goal of reducing HIV loads in blood and tissues by restoring the capacity of exhausted CD8+ cells to kill infected cells. This strategy consists of combining the IAP inhibitor D1143 with the ICI anti-PD-1 mAb in order to reinvigorate the exhausted immune system by PD-1 blockade.

To test the pre-clinical safety and efficacy of this strategy, we took advantage of the humanized BLT mouse model. This model is reliable across study groups (production of sufficient numbers of mice from a single tissue donor), and ideal to create group sizes that support strong statistical comparisons. We found that PD-1 expression on human CD8+ T cells increased during HIV infection, and that anti-PD-1 mAb treatment reduced it. This is in accordance with previous studies that showed that HIV infection elevates PD-1 expression on CD4+ and CD8+ T cells of infected humanized mice or individuals and that PD-1 blockade inhibits this effect [25–30, 55]. Importantly, D1143 intensifies the anti-PD-1 mAb-mediated reduction of PD-1 expression by CD8+ T cells. We found that D1143 enhances the anti-PD-1 mAb-mediated reduction in HIV loads in both blood and tissues including spleen, thymic organoid, lung, lymph nodes and liver. The D1143 enhancement of the anti-PD-1 effect on HIV load is more profound in organs than in blood. We found that only the anti-PD-1/D1143 combination could decrease HIV RNA levels in thymic organoid and lymph nodes at levels below the threshold of detection. Moreover, we obtained evidence that D1143 amplifies the anti-PD-1 mAb-mediated activation of effector functions of CD8+ T cells. Thus, there is a correlation between reduction of cell surface expression of PD-1 on CD8+ T cells, the reactivation of CD8+ T cell functions and the reduction in HIV loads in tissues by the anti-PD-1 treatment. Altogether these data suggest that the IAPa D1143 enhances the activation of CD8+ T cells and elimination of HIV by the anti-PD-1 mAb due to its co-stimulatory properties leading to enhanced CD8+ T cell activation. Therefore, the combination of D1143 with ICIs should improve the effects of immune checkpoint blockade and increase both cytopathic and immune-mediated reduction of HIV-infected cells in blood and tissues. Further studies should determine whether D1143 enhances the anti-HIV effects of all ICI members to a similar degree.

It remains to be fully understood how D1143 promotes the beneficial effects of the PD-1 blockade on the exhaustion reversal of CD8+ T cells for efficient HIV load reduction. One possibility is that D1143 enhances the effects of PD-1 blockade on CD8+ T cells by modulating the NF-kB response via degradation of specific components of the pathway as suggested previously [59]. Dougan et al. obtained evidence that IAPs play an important role in regulating T cell-dependent responses, suggesting that IAPa represent a strategy for developing novel immunomodulating therapies against cancer and chronic infections [59]. Another possibility is that D1143 induces direct or indirect apoptosis of infected cells, providing an explanation for the decrease in HIV cellular reservoirs in tissues. We will test this hypothesis in a subsequent D1143/anti-PD-1 humanized mouse experiment, by analyzing the *ex vivo* effect of D1143 on CD8+ T cells isolated from anti-PD-1 mAb-treated mice. Our finding that the PD-1-mediated decrease in HIV loads in blood by D1143 is not statistically significant while that in tissues is may arise from either distinct methodological procedure analyses or from a true biological phenomenon, indicating that further investigations are necessary to address these possibilities.

Our previous finding that D1143 reverses HIV latency by modulating the non-canonical NF-kB response [36], and our present finding that D1143 enhances PD-1 blockade effects on CD8+ T cells represent attractive properties for testing the combination of D1143 and anti-PD-1 mAb in a HIV latency model. Therefore, a similar study will be conducted in HIV-infected humanized mice under ART. The HIV latency model in BLT mice has been well described [60–61]. This approach should determine whether the properties of D1143 –HIV latency reversal and immunomodulation–represent a promising approach for the elimination of HIV reservoirs in blood and tissues.

## Supporting information

S1 Appendix Raw data for Fig 2. (TIF)

S2 Appendix Raw data for Fig 3. (TIF)

S3 Appendix Raw data for Fig 4. (TIF)

S4 Appendix Raw data for Fig 5. (TIF)

S5 Appendix Raw data for Fig 6. (TIF)
