# Supplementary figures and images for: The inhibitor of apoptosis proteins antagonist Debio 1143 promotes the PD-1 blockade-mediated HIV load reduction in blood and tissues of humanized mice
Source: PLoS One. 2020 Jan 24;15(1):e0227715. doi: 10.1371/journal.pone.0227715 (PMC6980394; doi:10.1371/journal.pone.0227715)

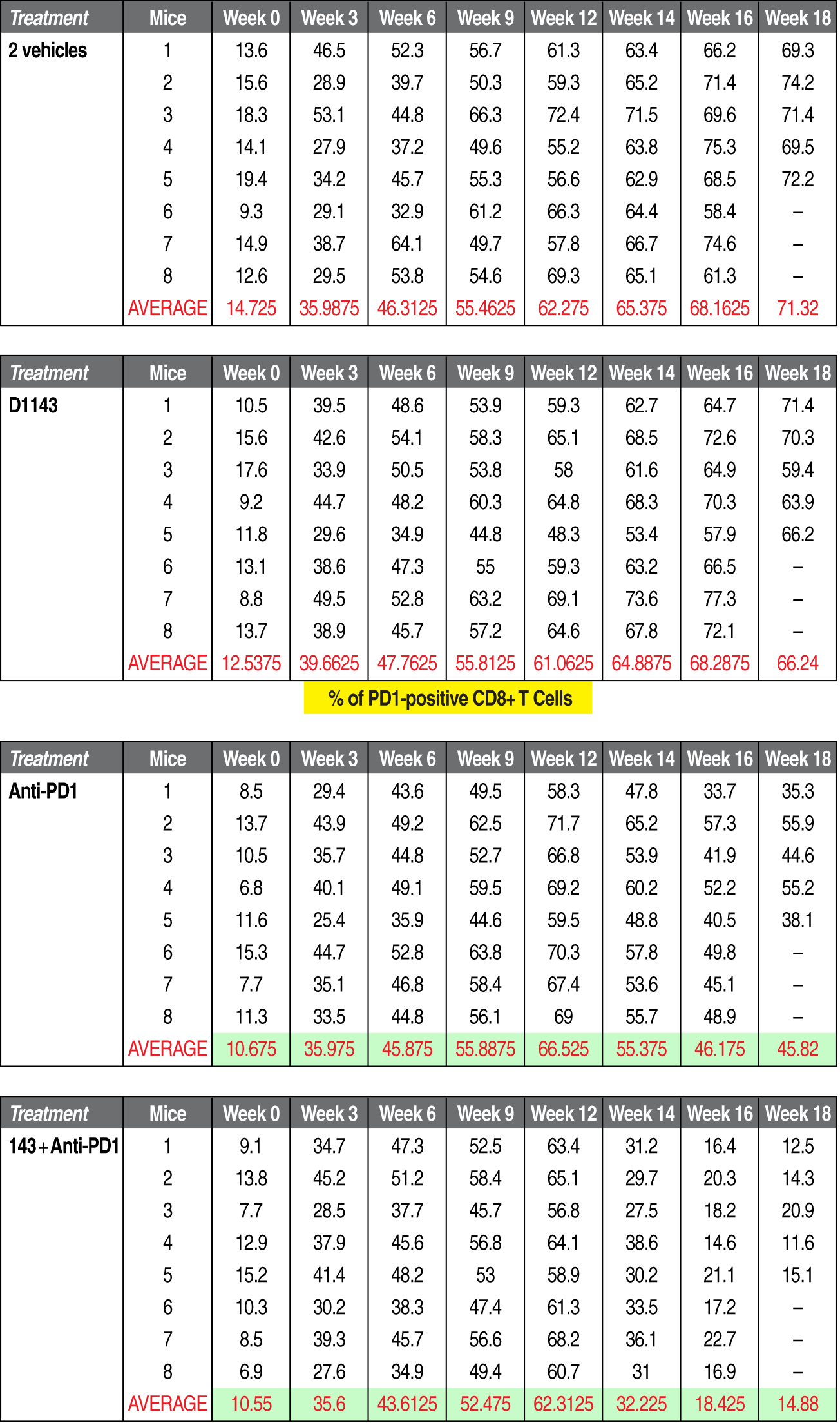

Supplement: S1 Appendix — (TIF) [file pone.0227715.s001.tif]

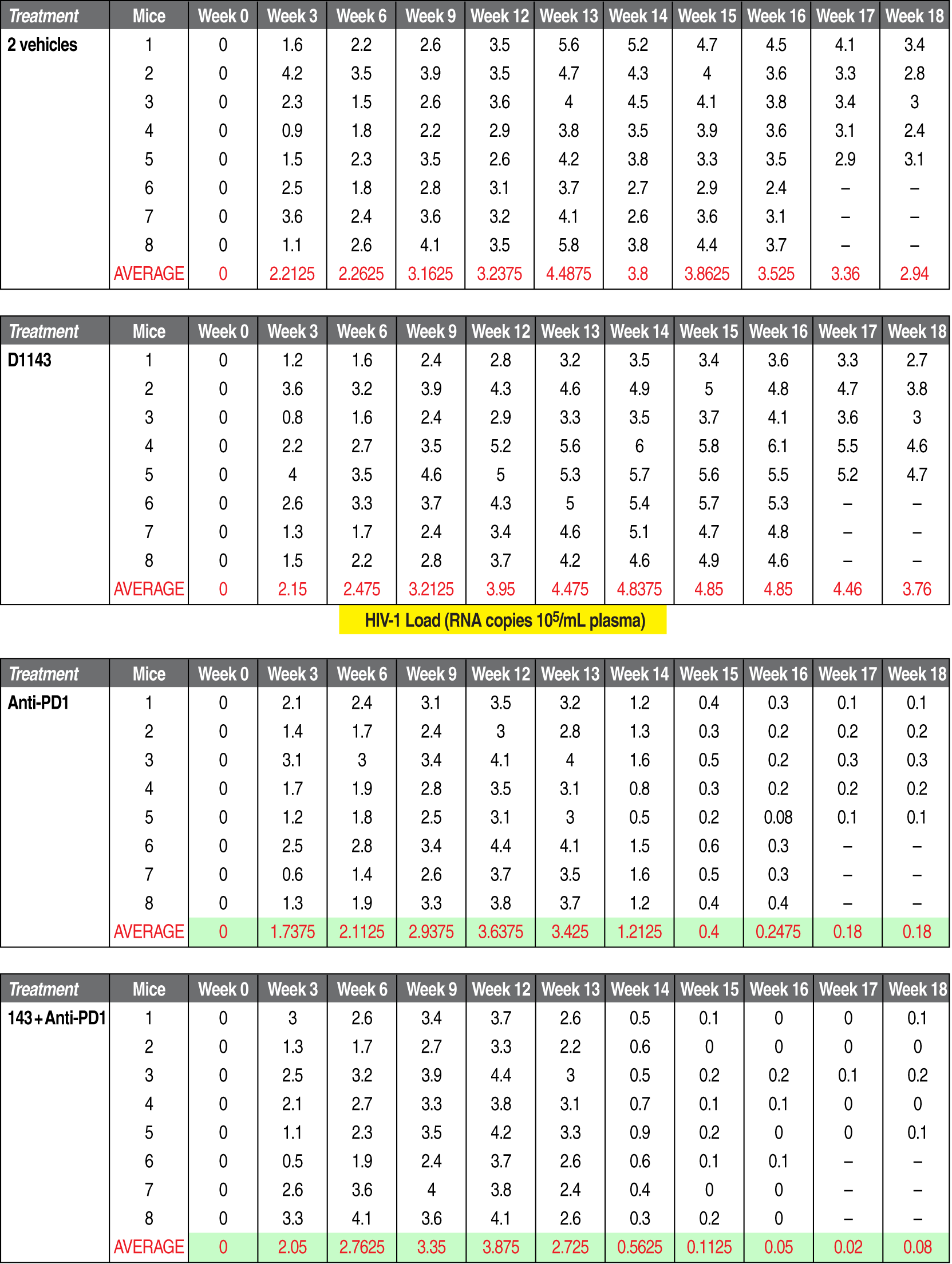

Supplement: S2 Appendix — (TIF) [file pone.0227715.s002.tif]

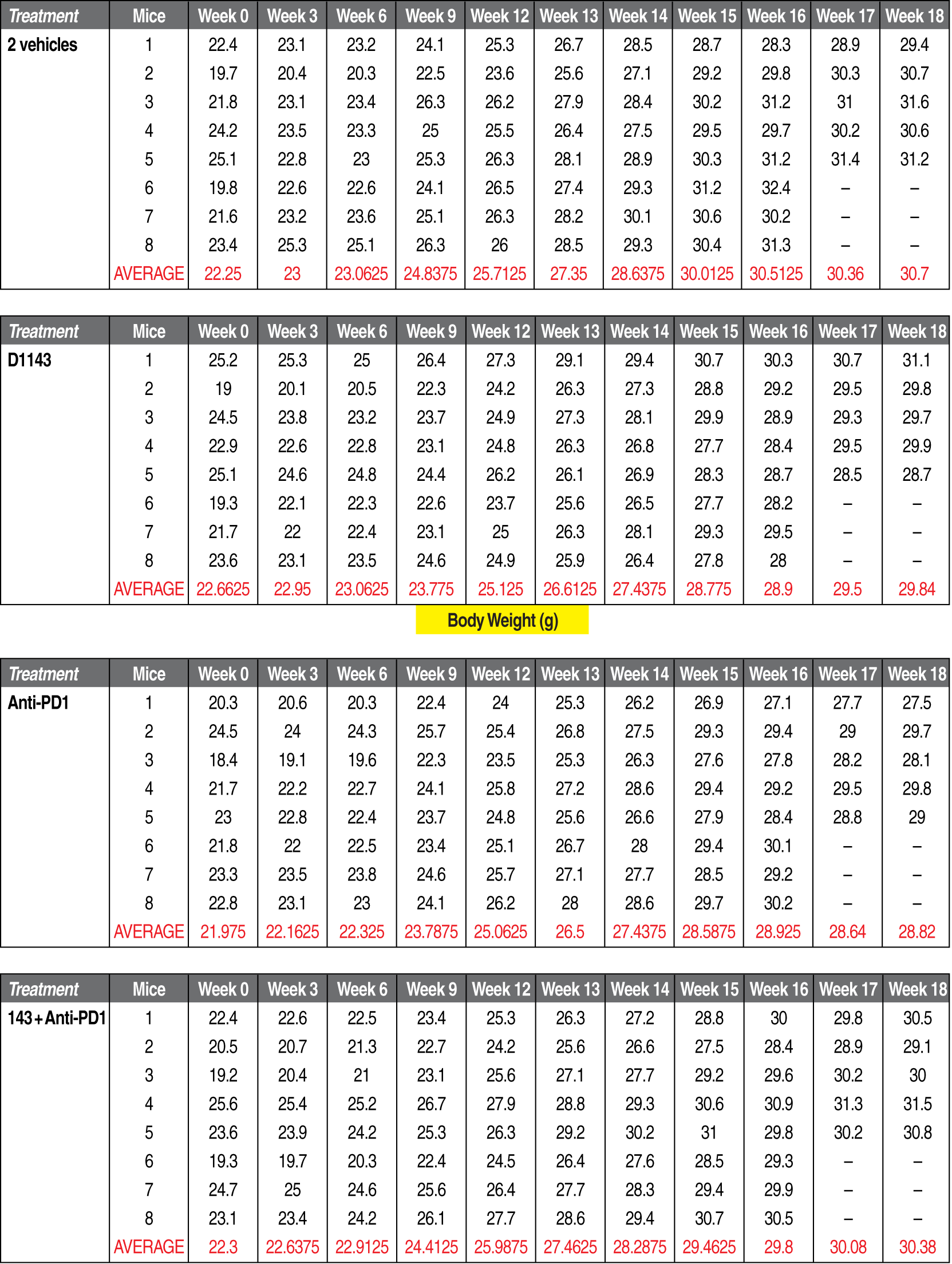

Supplement: S3 Appendix — (TIF) [file pone.0227715.s003.tif]

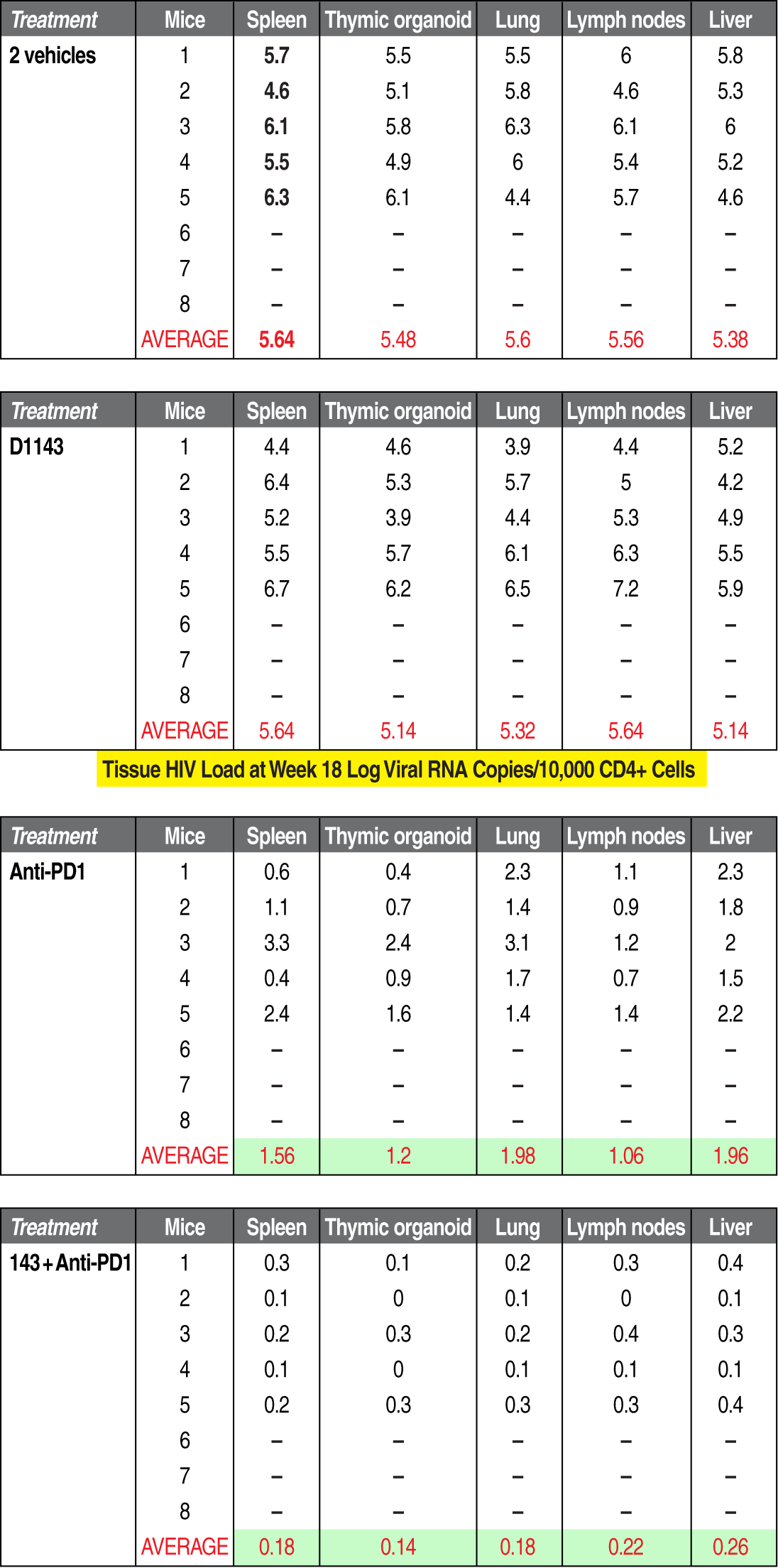

Supplement: S4 Appendix — (TIF) [file pone.0227715.s004.tif]

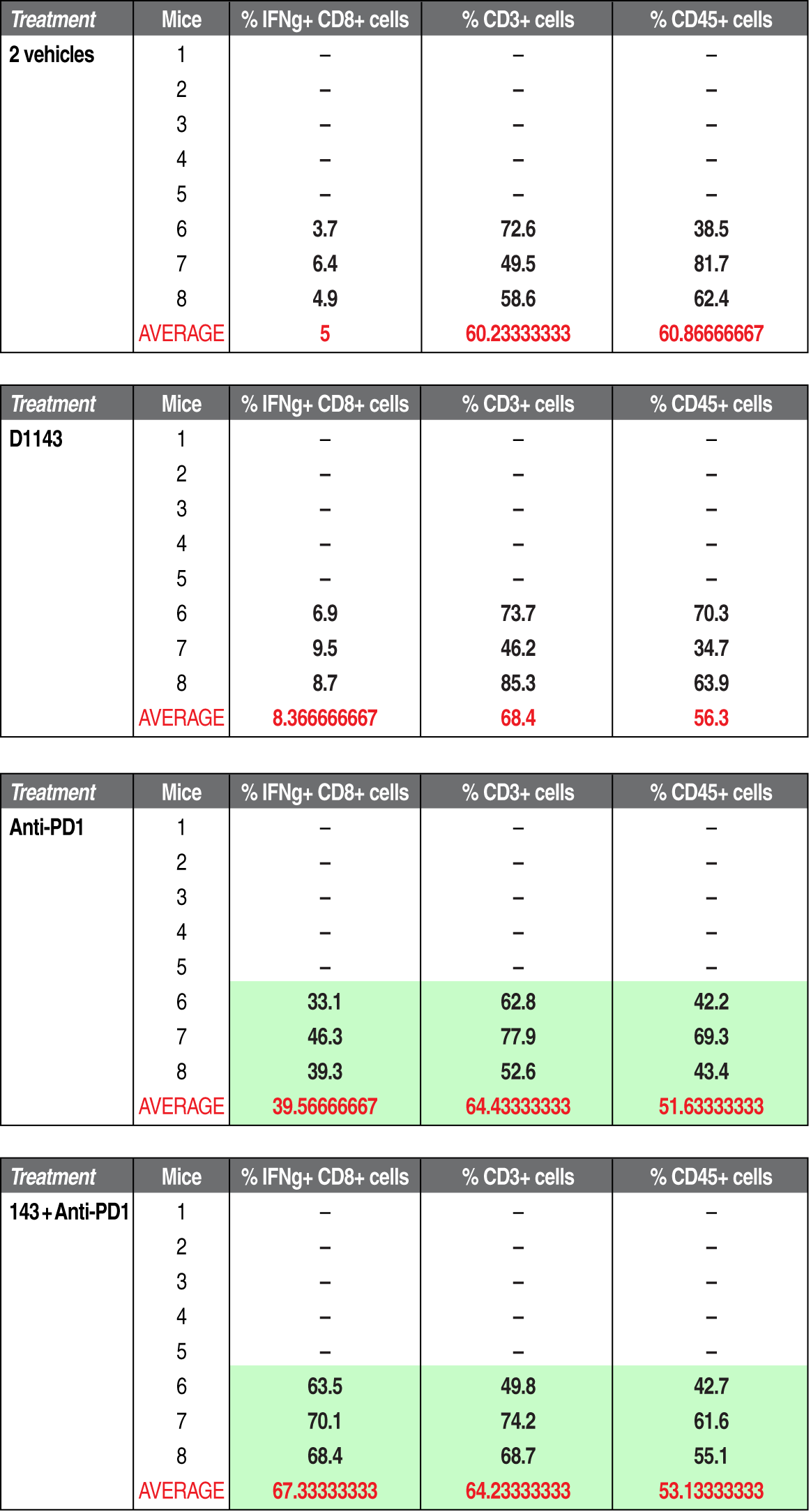

Supplement: S5 Appendix — (TIF) [file pone.0227715.s005.tif]
